# Low Grip Strength and Muscle Mass Increase the Prevalence of Osteopenia and Osteoporosis in Elderly Women

**DOI:** 10.3390/healthcare9040476

**Published:** 2021-04-16

**Authors:** Kyujin Lee, Ji Young Lee, Yong Hwan Kim

**Affiliations:** 1 Department of Physical Education, Institute of Sports Science, Seoul National University, Seoul 08826, Korea; my993286@snu.ac.kr; 2 Department of Physical Education, Gangneung-Wonju National University, Gangneung 25457, Korea

**Keywords:** grip strength, muscle mass, osteopenia, osteoporosis, sarcopenia, prevalence

## Abstract

The risk of developing low muscle strength and muscle mass is affected by aging, osteopenia, and osteoporosis and increases with age. The purpose of this study was to investigate the prevalence and cutoff values for osteoporosis and osteopenia according to the level of grip strength and muscle mass associated with sarcopenia. A cross-sectional study was conducted, and data from 734 women aged from 60 to 79 years old who visited the healthcare center from 2016 to 2019 were analyzed. Bone mineral density was measured on the lumbar spine from 1–4 using dual X-ray absorptiometry. Osteopenia and osteoporosis were classified on the basis of a T-score ranging from −1.0 to −2.4 and under −2.5, respectively. The diagnostic criteria for sarcopenia were a grip strength of <18 kg and muscle mass of <5.7 kg/m^2^ according to the Asian Working Group of Sarcopenia. Logistic regression analysis was used to determine the odds ratio, and the receiver operating characteristic curve was applied for the cutoff values. There were 351 (47.8%) patients with osteopenia and 152 (20.7%) patients with osteoporosis. The prevalence of osteopenia increased 1.593 times in the lowest grip strength group and 1.810 times in the lowest muscle mass group (*p* < 0.05). For osteoporosis, the lowest grip strength increased 2.512 times and the lowest muscle mass increased 2.875 times, compared to the highest grip strength group. In the sarcopenia group, osteopenia increased 2.451 times and osteoporosis increased 3.137 times, compared to the non-sarcopenia group (*p* < 0.05). In conclusion, the prevalence of osteoporosis and osteopenia was increased in elderly women with low grip strength and muscle mass.

## 1. Introduction

Osteopenia and osteoporosis are caused by a decreased rate of mineral deposition and bone remodeling, leading to weakened bone strength [1]. The typical method to measure bone mass and bone mineral density (BMD) is dual X-ray absorptiometry (DXA), with a T-score based on the number of standard deviations below the reference population (young adults) applied as a diagnostic criterion. Osteopenia and osteoporosis are diagnosed by a T-score of −1.0 to −2.4 and under −2.5, respectively [2]. Osteoporosis is reported to have a higher incidence in women than in men [3]. In the United States, the prevalence of osteoporosis is 14% in women over 50 years old, but only 6% in men in the same age group [4]. In addition, the BMD rapidly decreases in women after menopause [5]. Reduced BMD is dangerous because it causes an increased risk of osteoporotic fractures and a high mortality rate. In the United States, it has been reported that 1.5 million osteoporotic fractures occur annually. In particular, it is reported that approximately 31,000 people die within 6 months of hip fracture each year [6].

The causes of the decrease in BMD include both modifiable and nonmodifiable factors. Nonmodifiable risk factors for osteoporosis include age, sex, and heredity, while modifiable risk factors include lifestyle factors, such as physical activity, vitamin D supplementation, calcium intake, smoking cessation, and low alcohol consumption [4]. Furthermore, several studies have reported that low muscle strength and mass contribute to a high prevalence of low BMD and osteoporosis [7,8]. In a study of Asian individuals, the prevalence of lumbar osteoporosis increased to 3.481 in women with low grip strength compared to women with high grip strength [7]. Another study reported a 2.04-fold increase in the prevalence of total osteoporosis or osteopenia in people with low muscle mass [9].

Shin et al. [10] reported that the incidence of osteoporosis was 24.3% in women compared to 13.1% in men, and risk factors for vertebral osteoporosis in Korean women of over 40 age were related to monthly income and calcium intake. High monthly income had a decreased prevalence of osteoporosis by 0.64 compared to low monthly income, and the high calcium intake group had a decreased prevalence by 0.65 compared to the low calcium intake group.

Aging causes osteoporosis, but it also causes loss of muscle strength and muscle mass.

In 2016, this condition of low muscle strength and muscle mass state was named sacopenia and was assigned an International Classification of Diseases code (ICD 10-CM code M62.84) by the World Health Organization (WHO) [11]. Furthermore, according to the 2019 Asian Working Group of Sarcopenia (AWGS), the diagnostic criterion for sarcopenia in women require two conditions: muscle mass <5.7 kg/m^2^ and grip strength <18 kg [12]. Kwon et al. [13] reported that sarcopenia was 19.5% in women in their 50s, but increased to 22.1% in elderly women. Moreover, Lima et al. [14] found that osteoporosis had an incidence of 19.2% in pre-sarcopenia, but it increased to 35.3% in sarcopenia. Therefore, loss of muscle strength and muscle mass due to aging increases the risk of both sarcopenia and osteoporosis [15].

However, one of the debates about BMD is that the diagnosis of osteoporosis has different results depending on the site. In women over 50 years old, osteoporosis had an incidence of 11.6% in the femoral neck and 31.7% in the spine [10]. In Moayyeri et al.’s study [16], osteoporosis had an incidence of 12.4% in the total hip but increased to 24.7% in the spine. In the same study, this phenomenon was named discordance, and results revealed that major discordance occurred in 2.7% and minor discordance in 38.9% of cases.

Therefore, this study was conducted with the following characteristics: BMD was determined in the spine to reduce confusion according to the BMD measurement site. This study analyzed the prevalence of osteopenia and osteoporosis in women aged 60 to 79 years old with or without sarcopenia. Moreover, the cutoff values for grip strength and muscle mass for osteopenia and osteoporosis were calculated, and the prevalence of osteopenia and osteopenia was analyzed using cutoff values.

## 2. Methods

### 2.1. Participants

Data were obtained from women aged 60 to 79 who visited the healthcare screening center in Seoul, Korea from 2017 to 2018. Visits to the center are for the purpose of conducting preventative health management and associated tests, and they are generally not used to treat severe disease or for therapeutic purposes. Therefore, in the initially collected information, 4042 people met the age criteria of study, but women were excluded when they (1) had not completed the BMD measurement (*n* = 2849) and grip test or had measured grip strength in only one hand, (2) had diseases in the upper limbs including the hands, elbows, and shoulders within the last year (*n* = 154), (3) were currently taking medication or supplement for osteoporosis (*n* = 208), or (4) answered “no” to the question regarding whether they agreed to the use of their material for research purposes (*n* = 97). In addition, people with arthritis, stroke, and nervous system disorders at the level of disability considered to affect activity ability in the survey through the questionnaire were excluded from the data (*n* = 25). Data from 734 (18.1%) participants were ultimately included in the analysis. The examiner explained the purpose and methodology of the study to all participants; only the data of the women who gave consent were used in the analysis. This study was conducted in accordance with the guidelines of the Declaration of Helsinki for ethical research. This study was approved by the Institutional Review Board (AMC IRB, 2016-0084).

### 2.2. Test Procedure

Participants were asked to fast for 8 h, and all tests were conducted between 8 a.m. and 10 a.m. At the hospital, participants consulted with a doctor who checked for health problems on that day, and they completed questionnaires related to medical history, current medications, and socioeconomic status (e.g., monthly income and education level). The alcohol-related questions were surveyed in terms of weekly frequency, and the smoking status was described as ‘none’, ‘quit’, or ‘present’. For the frequency of exercise, aerobic and strength training were evaluated as follows: “Aerobic exercise; How many days a week do you do exercises that last longer than 20 min, such as walking, biking, running, or swimming?” “Strength training: How many days a week do you do exercises like dumbbells, strength equipment exercises, or sitting up with weight?”.

Participants were provided with light gowns and slippers for the examination by the organizer. Height, weight, and body composition were tested first, followed by grip strength and BMD measurements.

### 2.3. Bone Mineral Density

BMD was measured by the DXA method using Lunar Prodigy iDXA (General Electric Healthcare, Waukesha, WI, USA). Lumbar vertebrae L1–L4 were measured in the supine position, and BMD was recorded in g/cm^2^. The diagnostic criteria for osteopenia and osteoporosis were T-scores of −1.0 to −2.4 and ≤−2.5, respectively, according to WHO guidelines [10].

### 2.4. Sarcopenia

Sarcopenia was diagnosed by the presence of both low muscle strength and muscle mass according to the AWGS standard [12]. If only low muscle strength or muscle mass was present, the participant was diagnosed with pre-sarcopenia.

### 2.5. Grip Strength Test

Grip strength was measured using a grip strength dynamometer (TKK 5401, TAKEI, Niigata, Japan), with reference to the prior literature [17,18]. Prior to measurement, the participant performed stretching and light grip exercises, and the examiner provided an explanation and demonstration of the examination. The examiner adjusted the participant’s metacarpal phalangeal to be at right angles so that the sensor gauge fit the size of the participant’s hand.

During the testing, participants were directed to focus their eyes forward with legs shoulder-width apart and chest and waist straightened. The hand holding the device was in a neutral position with the hand lowered and the elbow extended such that the hand and machine did not touch the thigh. When the examiner gave a verbal signal as the “start”, the maximum grip was performed for approximately 2 s. During the examination, if the posture was disturbed or the hand touched the thigh, a retest was performed. After performing the test twice with each hand, the average maximum value was recorded in kg. In the current standard for sarcopenia, low muscle strength is defined as a hand grip of <18 kg for women [12].

### 2.6. Muscle Mass Measurement

Muscle mass was measured using bioelectrical impedance analysis (BIA) (Inbody 770, Inbody Inc., Seoul, Korea). For accurate measurement, the participant’s posture was extended with arms and legs apart such that the participants’ arms and thighs were not in contact with each other. After washing the hands and feet with alcohol or disinfectant wipes, the hands and feet were properly grounded with the machine. The muscle mass of the provided limb was used, and appendicular skeletal muscle mass (ASM) was calculated as weight (kg) divided by height squared (m^2^). The AWGS standard for sarcopenia is a muscle mass <5.7 kg/m^2^ for women [12].

### 2.7. Data Analysis 

Data analysis was performed using SPSS 25.0 (IBM SPSS, Armonk, NY, USA) for one-way ANOVA, chi-square test, and logistic regression analysis. Cutoff values were determined using receiver operating characteristic (ROC) curve analysis with MedCalc 17.9 (MedCalc Software, Ostend, Belgium). Continuous variables of general characteristics were expressed as the mean ± standard deviation; because age presented a significant difference in between groups, ANCOVA was performed including age in the covariate. Post hoc analysis was conducted using Bonferroni correction in the Kruskal–Wallis test The chi-square test was conducted to categorize BMD status according to sarcopenia factors and diagnosis.

In the data preprocessing, the data of people with missing values, input errors, and measurement errors, which were considered extreme values through a histogram, were deleted. Continuous variables excluding bone mass were set to one decimal place, and bone mass was set to three decimal places. The group setting of grip strength and muscle mass was classified by applying the quartile and sarcopenia criteria.

The prevalence of osteoporosis and osteopenia was determined by logistic regression analysis using the three methods. First, muscle strength and muscle mass were divided by quartile (Q), and participants were divided into the highest (Q1), high (Q2), low (Q3), and lowest (Q4) groups. Second, participants were grouped on the basis of the two criteria for sarcopenia: 18 kg of grip strength and 5.7 kg/m^2^ muscle mass. Participants were diagnosed with pre-sarcopenia if only one condition was present and with sarcopenia if both conditions were present. Third, a group was formed according to the cutoff value. Adjustment variables in model 1 included age and body weight, while those in model 2 included age, body weight, alcohol frequency, and strength exercise. Alcohol frequency and strength exercise were determined to be adjustment variables in model 2 on the basis of the results of the Chi-square test. The significance level was set at *p* < 0.05.

## 3. Results

According to the results of the study, the following findings were revealed: 31.5% of participants had normal BMD, 47.8% had osteopenia, and 20.7% had osteoporosis (Table 1). There were significant differences in age, height, weight, body mass index (BMI), bone mass, BMD, grip strength, and muscle mass (*p* < 0.05). In particular, differences between participants with osteopenia and osteoporosis were found in age, bone mass, BMD, grip strength, and muscle mass (*p* < 0.05). A grip strength of under 18 kg had an incidence of 13.9% in normal BMD, 17.1% in osteopenia, and 21.1% in osteoporosis (*p* = 0.033). Muscle mass at the level of sarcopenia had an incidence of 9.1% in normal BMD, 21.9% in osteopenia, and 31.6% in osteoporosis. Low muscle strength and muscle mass were associated with low BMD (*p* < 0.001).

There were significant differences in grip strength (*p* < 0.001) and muscle mass (*p* < 0.001) between groups. Specifically, grip strength and muscle mass were significantly lower in osteopenia and osteoporosis than in normal BMD, and they were significantly lower in osteoporosis than in osteopenia. The incidence of osteopenia and osteoporosis according to the criteria for sarcopenia is presented in Table 1. Sarcopenia had an incidence of 7.4% in normal BMD, 8.0% in osteopenia, and 14.5% in osteoporosis (*p* < 0.05). In the analysis of socioeconomic status, there was significance between groups in monthly income (*p* = 0.016), but not in education level (*p* = 0.107).

Table 2 showed the chi-square test for health behavior according to the groups. The rows were arranged in the normal BMD, osteopenia, and osteoporosis groups, and the columns were classified by categorizing health behaviors such as alcohol frequency, smoking status, and aerobic and strength exercise frequency. There was no significant result of smoking status between groups (*p* = 0.072). The proportion of people who did not smoke at all was very high (93.0% in normal BMD, 90.6% in sarcopenia, and 90.1% in osteoporosis). There were significant differences between groups in terms of alcohol frequency (*p* < 0.001). The osteoporosis group showed a result of 5.9% at 2–3 days per week and 4.6% at 4–7 days, which was higher than that of other groups. In the exercise habit survey, there was no significant difference in aerobic exercise (*p* = 0.124), but the difference in strength exercise was significant (*p* = 0.021).

Table 3 and Table 4 show the odds ratios for osteopenia according to sarcopenia. Osteopenia was increased 1.593-fold (Model 2, 95% CI 1.109–3.393, *p* < 0.001) in the group with the lowest grip strength and 1.810-fold (Model 2, 95% CI 1.649–4.031, *p* < 0.001) in the group with the lowest muscle mass. The sarcopenia group showed a 2.451-fold increase in osteopenia. Osteoporosis increased 2.512-fold (Model 2, 95% CI 1.569–5.644, *p* < 0.001) and 2.875-fold (Model 2, 95% CI 1.803–6.854, *p* < 0.001) in the groups with the lowest grip strength and muscle mass, respectively. In addition, the group with sarcopenia had a 3.137-fold (Model 2, 95% CI 1.799–7.036, *p* < 0.001) increase in osteoporosis.

**Figure 1:**
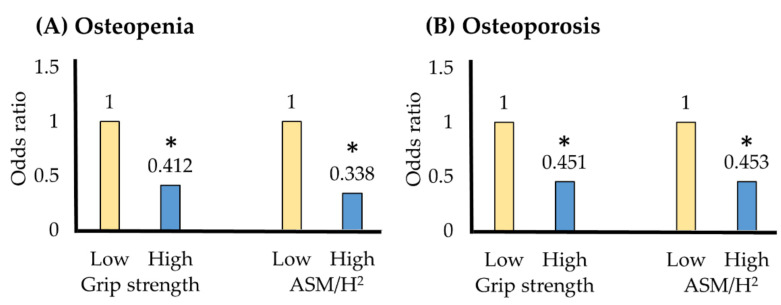
(**A**) Osteopenia and (**B**) osteoporosis odds ratios according to cutoff values. * *p* < 0.05; OR = odds ratio; CI = confidence interval; ASM/H^2^ = appendicular skeletal muscle mass/height^2^. Model 1 = adjustment variables age, body weight. Model 2 = adjustment variables age, body weight, alcohol frequency, strength exercise.

Table 5 shows the grip strength and muscle mass cutoff values for osteopenia and osteoporosis using the ROC curve. The grip strength cutoff for osteoporosis was 23.1 kg; the prevalence of osteoporosis decreased to 0.451 in the group with a grip strength above this cutoff value (Figure 1). The muscle mass cut-off for osteoporosis was 6.5 kg/m^2^; the prevalence of osteoporosis was reduced to 0.453 in the group with muscle mass greater than the cutoff value.

## 4. Discussion

Decreased muscle strength, muscle mass, and BMD are typical physiological changes associated with aging [19]. Low muscle mass and muscle strength are diagnostic criteria for sarcopenia and are among the risk factors associated with decreased BMD [15]. Therefore, this study analyzed the effect of sarcopenia on the prevalence of osteopenia and osteoporosis using a cross-sectional design.

In this study, the prevalence of osteopenia and osteoporosis was 47.8% and 20.7%, respectively, according to BMD measurements of the spine. These results are similar to prior reports indicating that the prevalence of spinal osteoporosis was 24.0% in Korean women over 50 years old [10] and 24.7% in a Japanese cohort [16]. However, the prevalence of osteoporosis varies depending on the measurement site. For example, the prevalence of osteoporosis decreased to 5.7% when measured at the femoral neck in a Korean study [10], while it was only 12.4% when measured at the total hip in a Japanese study [16]. The prevalence of osteoporosis also varies geographically. In a study of Chinese women, osteoporosis was 11.8% in Beijing, but 24.5% in Jilin. In the same study, the prevalence of osteoporosis in women over 50 years of age, even of similar races, was 34.1% to 37.0% in Hong Kong but only 11.4% in Taiwan [20]. This geographic variability may stem from the fact that modifiable factors, such as physical activity, vitamin D deficiency, urban and local environment, daily lifestyle, health status, and accessibility, affect osteoporosis in addition to the nonmodifiable factors of gender, race, and age [2,20,21].

In this study, sarcopenia was found in 67 of 734 (9.1%) participants, which is a relatively low prevalence. The prevalence of sarcopenia in Korean women in their 60s and 70s was previously reported to be 16.6% and 23.7%, respectively [13]. In Japanese women in their 60s and 70s, the prevalence of sarcopenia was 14.2% and 14.9%, respectively [22]. In a meta-analysis, between 8% to 40% of the individuals over 60 years old reported sarcopenia [23]. In a review study that analyzed the studies of Asian countries, the prevalence of sarcopenia varied from 6.7% to 56.7% in men and 0.1% to 33.6% in women [24]. The large variation in prevalence rates between studies is attributed to a lack of standard criteria for sarcopenia. While the WHO standards for osteoporosis have been applied globally for many years [25], different standards for sarcopenia have been set forth by the AWGS, the European Working Group on Sarcopenia in Older People (EWGSOP), and the Foundation for the National Institutes of Health Sarcopenia Project (FNIH) [12,26,27]. For example, in the case of excitation grip strength, the cutoff values established by the FNIH and AWGS are 16 kg and 18 kg, respectively [12,26]. In addition, for men, the AWGS criterion of grip strength was 26 kg in 2016, but it changed to 28 kg in 2019 [12]. The same organization may also suggest different standards depending on the measuring equipment used. For example, the AWGS threshold for low muscle mass is 5.4 kg/m^2^ when measured by DXA, but 5.7 kg/m^2^ when measured by BIA. Thus, prevalence rates may vary even within the same population, depending on the measurement tool used. When measuring 250 elderly people over 65 years old, the prevalence of sarcopenia was 8.4% to 27.6% with DXA and 8.4% to 17.2% with BIA [28]. One factor that may have influenced the low prevalence of sarcopenia was the socioeconomic status of the participants. Our study likely included many people with high socioeconomic status because healthcare centers in private hospitals are relatively expensive. These individuals are more likely to be in good health, be physically active, and have a positive nutritional status [29,30].

A primary finding of this study is that the prevalence of osteopenia and osteoporosis increased as muscle strength decreased. These results are consistent with those of previous studies. In a study of 500 women aged 60–85 years, individuals with sarcopenia had a 2.515-fold increase in the prevalence of osteoporosis compared to healthy participants [14]. Similarly, another study found that the prevalence of osteoporosis in people with sarcopenia increased 7.3-fold [31]. Conversely, in people with osteopenia and osteoporosis, the rate of sarcopenia is high. In a study of 2400 Japanese women, 16.8% of those with osteopenia and 20.4% with osteoporosis also had sarcopenia [22]. Thus, the causality of these two diseases is still controversial.

This study sought to determine the prevalence of osteoporosis and osteopenia according to grip strength and muscle mass, respectively. While several studies have reported a significant correlation, few have analyzed the odds ratio. Most studies reported low BMD in people with low grip strength through correlation and single or multiple regression analyses [7,32,33]. However, there have also been studies showing different results. In one study, BMD was related to muscle mass, but it was not significantly related to grip strength [34]. In another study, grip strength was significantly lower in women with osteoporosis, but no significant increase in odds ratio was found [35]. However, more studies have reported a significant association. A study of grip strength and BMD in 120 women showed that the prevalence of osteoporosis increased in the low grip strength group 4.138-fold in the total hip, 5.744-fold in the femoral neck, and 3.481-fold in the lumbar spine [7]. In addition, the number of vertebral fractures increased 2.67-fold in women with low grip strength [35]. Osteoporosis is dangerous in the elderly because it also affects the mortality rate. In a study that followed 6565 people for 22 years, the mortality rate increased 1.13- and 1.17-fold in men and women, respectively, with osteopenia, and 1.37- and 1.32-fold in men and women, respectively, with osteoporosis, even when adjusting for muscle strength, chronic disease, physical activity, and education level [36].

Grip strength has long been studied in relation to aging [37]. Grip strength is a simple, safe, and inexpensive measurement with high reliability and validity [38]. In particular, many studies have shown that grip strength is related to cardiovascular disease or metabolic syndrome [39,40,41].

An important aspect of this study was that the cutoff values were calculated using the ROC curve. The cutoff values for grip strength were 23.1 kg in osteoporosis and 22.6 kg in osteopenia. These values are slightly higher than the AWGS guideline of 18 kg and can be applied as a value required for bone health.

Methods for measuring muscle mass include imaging such as MRI, CT, and DXA, and BIA using electrical signals [42,43]. The imaging method has the advantage of measuring the actual amount, but it requires a lot of time and cost. This is the reason why a body composition test using DXA was not possible herein, and this is a limitation of this study. Meanwhile, BIA is simple and relatively inexpensive, while it has high reliability and validity compared to DXA. In a previous study, Inbody 770 equipment showed high reliability (standard error of the measurement, 0.58 kg) and correlation compared to DXA [44,45].

Sarcopenia was designated as a disease by the WHO in 2016 and must be managed, especially for the elderly [11]. Therefore, the results presented in this study have clinical significance in that they propose reference values for grip strength and muscle mass to prevent osteoporosis and osteopenia. However, a limitation of this study is its inability to confirm the causality of sarcopenia and osteoporosis due to the cross-sectional design. In addition, an increase in muscle mass was proportional to an increase in weight. Therefore, researchers argue that sarcopenic obesity should be studied rather than sarcopenia alone [46]. Moreover, since the study population did not include men and only included those who participated in the healthcare center, the study outcome cannot be generalized. BMD peaks in the early 30s and is influenced by factors such as exercise and proper nutrition [47,48]; however, nutritional status was not included in this study. In individuals with sarcopenia, muscle function can be evaluated by measuring gait speed in addition to grip strength [12]; however, gait speed was not measured in this study. In the future, there is a need for longitudinal studies. Furthermore, it will be necessary to experimentally confirm the preventive effect of reducing BMD through strength training and muscle mass. In this study, the type and frequency of exercise were investigated, but the time and intensity were not included. Neither muscle strength nor cardiopulmonary endurance was included. It will be meaningful to compare physical fitness and exercise habits in BMD in future studies. The literature highlights a difference in BMD according to the characteristics of residential areas such as cities and localities [20,49]. In this study, participants visited from various regions across the country, but the researchers did not consider the residential area in the study, which is an additional limitation of this study. In addition, since the sample size was too small to analyze by region, it will be necessary to investigate the residential area and characteristics of more participants in future studies. Lastly, BMD shows different results depending on the measurement site [16]. Only 58.3% of the results were consistent. Therefore, further studies are needed to analyze the difference between sarcopenia and relevance by analyzing the BMD of various sites.

## 5. Conclusions

Sarcopenia increased osteopenia 2.1-fold and osteoporosis 3.1-fold. The prevalence of osteoporosis was decreased in elderly women with cutoff values of 23.1 kg for grip strength and 6.5 kg/m^2^ for muscle mass. Therefore, low grip strength and low muscle mass increased the prevalence of osteoporosis and osteopenia in elderly women. 

## Figures and Tables

**Table 1:** Characteristic of participants.

Variables	Normal BMD	Osteopenia	Osteoporosis	*p*
*n* (%)	231 (31.5%)	351 (47.8%)	152 (20.7%)	
Age, years	64.3 ± 4.0	65.3 ± 4.7 ^a^	67.2 ± 4.1 ^b,c^	<0.001 *
Height, cm	158.2 ± 5.0	156.1 ± 3.9 ^a^	156.3 ± 4.6 ^b^	<0.001 *
Weight, kg	61.8 ± 9.4	55.4 ± 5.9 ^a^	55.8 ± 6.7 ^b^	<0.001 *
BMI, kg/m^2^	24.7 ± 3.9	22.7 ± 2.1 ^a^	22.8 ± 2.6 ^b^	<0.001 *
Bone mass, g/cm^2^	1.147 ± 0.132	0.934 ± 0.060 ^a^	0.807 ± 0.047 ^b,c^	<0.001 *
Bone mineral density, T-score	0.4 ± 1.1	−1.8 ± 0.5 ^a^	−2.8 ± 0.4 ^b,c^	<0.001 *
Grip strength, kg	23.8 ± 4.6	21.3 ± 4.2 ^a^	19.2 ± 4.2 ^b,c^	<0.001 *
≥18 kg, *n* (%)	199 (86.1%)	291 (82.9%)	120 (78.9%)	0.033 *
<18 kg, *n* (%)	32 (13.9%)	60 (17.1%)	32 (21.1%)	
Muscle mass, ASM/H^2^	6.6 ± 0.7	6.2 ± 0.4 ^a^	5.9 ± 0.5 ^b,c^	<0.001 *
≥5.7 kg/m^2^, *n* (%)	210 (90.9%)	274 (78.1%)	104 (68.4%)	<0.001 *
<5.7 kg/m^2^, *n* (%)	21 (9.1%)	77 (21.9%)	48 (31.6%)	
Sarcopenia diagnosis				
Non-sarcopenia	171 (74.0%)	201 (57.2%)	71 (46.7%)	<0.001 *
Pre-sarcopenia	43 (18.6%)	122 (34.8%)	59 (38.8%)	
Sarcopenia	17 (7.4%)	28 (8.0%)	22 (14.5%)	
Monthly income, KRW				
>7,000,000	44 (19.0%)	62 (17.7%)	20 (13.1%)	0.016 *
5,000,000 to 7,000,000	115 (49.8%)	152 (43.3%)	58 (38.2%)	
<5,000,000	72 (31.2%)	137 (39.0%)	74 (48.7%)	
Education level				
To graduate school	26 (11.2%)	32 (9.2%)	11 (7.3%)	0.107 *
To college	84 (36.4%)	116 (33.0%)	40 (26.3%)	
To high school or under	121 (52.4%)	203 (57.8%)	101 (66.4%)	

* *p* < 0.05; values are expressed as the mean ± standard deviation or *n* (%); Kruskal–Wallis test and chi-square test were performed for comparison between groups. ^a^ = Normal BMD vs. osteopenia; ^b^ = normal BMD vs. osteoporosis; ^c^ = osteopenia vs. osteoporosis; BMD = bone mineral density; BMI = body mass index; ASM/H^2^ = appendicular skeletal muscle mass/height^2^; KRW = Korean won.

**Table 2:** Health behavior according to normal BMD, osteopenia, and osteoporosis.

Variables	Classification	Normal BMD (*n* = 231)	Osteopenia (*n* = 351)	Osteoporosis (*n* = 152)	*p*
Smoking status, (%)	None	215 (93.0%)	318 (90.6%)	137 (90.1%)	0.072
	Quit	8 (3.5%)	21 (6.0%)	11 (7.3%)	
	Present	8 (3.5%)	12 (3.4%)	4 (2.6%)	
Alcohol frequency, (%)	None	135 (58.5%)	213 (60.7%)	92 (60.5%)	<0.001 *
	1 day/month	58 (25.1%)	79 (22.5%)	30 (19.7%)	
	2–4 days/month	27 (11.7%)	38 (10.8%)	14 (9.2%)	
	2–3 days/week	7 (3.0%)	8 (2.3%)	9 (5.9%)	
	4–7 days/week	4 (1.7%)	13 (3.7%)	7 (4.6%)	
Aerobic exercise frequency, (%)	None	30 (13.0%)	60 (17.1%)	39 (25.7%)	0.124
	1–2 days/week	71 (30.7%)	110 (31.3%)	49 (32.2%)	
	3–4 days/week	83 (35.9%)	120 (31.2%)	42 (27.6%)	
	5–7 days/week	47 (20.3%)	61 (17.4%)	22 (14.5%)	
Strength exercise frequency, (%)	None	125 (54.1%)	205 (58.4%)	98 (64.5%)	0.021 *
	1–2 days/week	27 (11.7%)	77 (21.9%)	31 (20.4%)	
	3–4 days/week	61 (26.4%)	56 (16.0%)	19 (12.5%)	
	5–7 days/week	18 (7.8%)	13 (3.7%)	4 (2.6%)	

* *p* < 0.05; values are expressed as numbers and percentages; BMD = bone mineral density.

**Table 3:** Odds ratio of osteopenia according to grip strength and muscle mass.

Variables	Group	Model 1		Model 2	
		OR (95% CI)	*p*	OR (95% CI)	*p*
Grip strength	Q1	1	-	1	-
	Q2	1.008 (0.609–2.164)	0.144	1.024 (0.796–2.433)	0.124
	Q3	1.052 (0.855–2.622)	0.156	1.491 (0.990–3.531)	0.101
	Q4	1.443 (1.048–5.407)	<0.001 *	1.593 (1.109–3.393)	<0.001 *
Muscle mass	Q1	1	-	1	-
	Q2	1.018 (0.793–2.096)	0.251	1.176 (0.849–2.926)	0.150
	Q3	1.343 (0.936–3.603)	0.235	1.220 (1.061–4.551)	0.201
	Q4	1.521 (1.177–4.130)	<0.001 *	1.810 (1.649–4.031)	<0.001 *
Grip strength	≥18 kg	1	-	1	-
	<18 kg	1.115 (1.015–3.125)	0.027 *	1.280 (1.114–4.159)	<0.001 *
Muscle mass	≥5.7 kg/m^2^	1	-	1	-
	<5.7 kg/m^2^	1.185 (1.125–4.874)	<0.001 *	1.810 (2.037–5.767)	0.021 *
Sarcopenia	Nonsarcopenia	1	-	1	-
	Presarcopenia	1.710 (1.022–3.874)	0.030 *	2.010 (1.015–4.141)	0.004 *
	Sarcopenia	2.011 (1.249–4.057)	<0.001 *	2.451 (1.112–5.254)	0.011 *

* *p* <0.05; OR = odds ratio; CI = confidence interval; Model 1 = adjustment variables: age and body weight. Model 2 = adjustment variables: age, body weight, alcohol frequency, and strength exercise.

**Table 4:** Odds ratio of osteoporosis according to grip strength and muscle mass.

Variables	Group	Model 1		Model 2	
		OR (95%CI)	*p*	OR (95%CI)	*p*
Grip strength	Q1	1	-	1	-
	Q2	1.011 (0.777–3.631)	0.254	1.869 (1.091–4.402)	0.421
	Q3	1.716 (1.199–4.337)	0.322	1.240 (1.372–4.546)	0.011 *
	Q4	2.265 (1.851–5.742)	<0.001 *	2.512 (1.569–5.644)	<0.001 *
Muscle mass	Q1	1	-	1	
	Q2	1.024 (0.431–2.436)	0.557	1.740 (1.019–4.172)	0.043 *
	Q3	1.805 (1.359–5.780)	0.002 *	1.278 (1.058–4.820)	0.026 *
	Q4	2.073 (1.123–6.358)	0.026 *	2.875 (1.803–6.854)	<0.001 *
Grip strength	≥18 kg	1	-	1	-
	<18 kg	1.259 (1.010–3.505)	0.008 *	1.334 (1.007–2.886)	0.032 *
Muscle mass	≥5.7 kg/m^2^	1	-	1	-
	<5.7 kg/m^2^	2.097 (1.035–5.895)	0.040 *	2.268 (1.532–8.357)	<0.001 *
Sarcopenia	Non-sarcopenia	1	-	1	-
	Pre-sarcopenia	2.600 (1.058–5.045)	0.012 *	2.812 (1.155–6.845)	0.005 *
	Sarcopenia	2.508 (1.145–5.845)	<0.001 *	3.137 (1.799–7.036)	<0.001 *

* *p* < 0.05; OR = odds ratio; CI = confidence interval. Model 1 = adjustment variables: age and body weight. Model 2 = adjustment variables: age, body weight, alcohol frequency, and strength exercise.

**Table 5:** Cutoff values of grip strength and muscle mass for osteopenia and osteoporosis.

Variables	Cut-Off	AUC (95% CI)	Sensitivity	Specificity	*p*
Grip strength					
Osteopenia	22.6 kg	0.595 (0.325–0.894)	90.5	32.6	0.017 *
Osteoporosis	23.1 kg	0.593 (0.312–0.912)	85.7	40.4	0.022 *
ASM/H^2^					
Osteopenia	6.4 kg/m^2^	0.705 (0.428–0.974)	81.1	53.3	0.016 *
Osteoporosis	6.5 kg/m^2^	0.587 (0.287–0.891)	76.2	41.1	0.022 *

* *p* < 0.05; AUC = area under the curve; CI = confidence interval; ASM/H^2^ = appendicular skeletal muscle mass/height^2^.

## Data Availability

The data are not publicly available due to privacy or ethical reasons.
